# Revealing the potential therapeutic mechanism of *Lonicerae Japonicae Flos* in Alzheimer’s disease: a computational biology approach

**DOI:** 10.3389/fmed.2024.1468561

**Published:** 2024-11-13

**Authors:** Qin Xiang, Yu Xiang, Yao Liu, Yongjun Chen, Qi He, Taolin Chen, Liang Tang, Binsheng He, Jianming Li

**Affiliations:** ^1^Hunan Provincial University Key Laboratory of the Fundamental and Clinical Research on Neurodegenerative Diseases, Changsha Medical University, Changsha, China; ^2^Hunan Provincial University Key Laboratory of the Fundamental and Clinical Research on Functional Nucleic Acid, Changsha Medical University, Changsha, China; ^3^Hunan Provincial Key Laboratory of the Traditional Chinese Medicine Agricultural Biogenomics, Changsha Medical University, Changsha, China; ^4^College of Basic Medicine, Changsha Medical University, Changsha, China; ^5^Department of Neurology, Nanhua Affiliated Hospital, Hengyang Medical College, University of South China, Hengyang, China; ^6^Ziyang District Brain Hospital, Yiyang, China

**Keywords:** Alzheimer’s disease, computational biology, *Lonicerae Japonicae Flos*, mechanisms, traditional Chinese medicine

## Abstract

**Background:**

Alzheimer’s disease (AD) is a degenerative brain disease without a cure. *Lonicerae Japonicae Flos* (LJF), a traditional Chinese herbal medicine, possesses a neuroprotective effect, but its mechanisms for AD are not well understood. This study aimed to investigate potential targets and constituents of LJF against AD.

**Methods:**

Network pharmacology and bioinformatics analyses were performed to screen potential compounds and targets. Gene Expression Omnibus (GEO) datasets related to AD patients were used to screen core targets of differential expression. Gene expression profiling interactive analysis (GEPIA) was used to validate the correlation between core target genes and major causative genes of AD. The receiver operating characteristic (ROC) analysis was used to evaluate the predictive efficacy of core targets based on GEO datasets. Molecular docking and dynamics simulation were conducted to analyze the binding affinities of effective compounds with core targets.

**Results:**

Network pharmacology analysis showed that 112 intersection targets were identified. Bioinformatics analysis displayed that 32 putative core targets were identified from 112 intersection targets. Only eight core targets were differentially expressed based on GEO datasets. Finally, six core targets of MAPK8, CTNNB1, NFKB1, EGFR, BCL2, and NFE2L2 were related to AD progression and had good predictive ability based on correlation and ROC analyses. Molecular docking and dynamics simulation analyses elucidated that the component of lignan interacted with EGFR, the component of β-carotene interacted with CTNNB1 and BCL2, the component of β-sitosterol interacted with BCL2, the component of hederagenin interacted with NFKB1, the component of berberine interacted with EGFR and BCL2, and the component of baicalein interacted with NFKB1, EGFR and BCL2.

**Conclusion:**

Through a comprehensive analysis, this study revealed that six core targets (MAPK8, CTNNB1, NFKB1, EGFR, BCL2, and NFE2L2) and six practical components (lignan, β-carotene, β-sitosterol, hederagenin, berberine, and baicalein) were involved in the mechanism of action of LJF against AD. Our work demonstrated that LJF effectively treats AD through its multi-component and multi-target properties. The findings of this study will establish a theoretical basis for the expanded application of LJF in AD treatment and, hopefully, can guide more advanced experimental research in the future.

## 1 Introduction

Alzheimer’s disease (AD) is the predominant neurological disease among the elderly population, recognized as the primary cause of dementia, and emerging as a significant public health concern with global implications (1). AD is characterized clinically by a progressive deterioration of cognitive functions and a gradual memory decline (2). Neuropathologically, AD is featured by significant cortical atrophy, ventricular enlargement, extracellular amyloid deposition, and intracellular neurofibrillary tangles composed of hyperphosphorylated tau proteins (1, 3). Research findings indicate that genetic risk factors associated with AD account for 60–80% of the overall risk (4). Presently, the pharmaceutical agents utilized in the treatment of AD, including donepezil, galantamine, rivastigmine and memantine, are characterized by their single-target mechanisms of action, which have demonstrated some efficacy in managing the symptoms of the disease (5, 6). However, due to the clinical and genetic heterogeneity of AD, the effectiveness and safety of single-target drugs have yet to meet the anticipated outcomes. It is widely believed that multi-targeted drugs can target multiple factors within the disease network, leading to enhanced effect and reduced occurrence of side effects. Therefore, the development of multi-target drugs for the treatment of AD has emerged as a major focus of research in recent years to tackle these challenges.

Traditional Chinese medicinal herbs are well-suited for drug discovery in prolonged AD treatment (7, 8). *Herba Epimedii*, *Coptis Chinensis Franch*, *Rhizoma Curcumae Longae*, *Green tea*, *Ganoderma*, and *Panax Ginseng* have shown significant potential as herbal candidates for developing effective anti-AD medications (6, 9). *Lonicerae Japonicae Flos* (LJF) has been extensively applied to traditional Chinese medicine for numerous centuries as a medicinal remedy, demonstrating established therapeutic properties (10). LJF was initially documented in Li Shizhen’s “Compendium of Materia Medica” and incorporated into the Chinese Pharmacopoeia (11). According to the principles of Chinese medicine, LJF is believed to possess the capabilities of heat-clearing and detoxifying, as well as the dispersion of wind-heat, among other functions (12). Currently, over 300 compounds, including volatile oils, organic acids, and flavonoids, have been found in LJF extract (13). These compounds demonstrate various beneficial properties, such as antiviral, anti-inflammatory, anti-tumor, antioxidant, and immune regulatory effects (13). Pharmacological studies have shown that LJF water extracts have the potential to be therapeutically effective in treating Huntington’s disease, Parkinson’s disease, and AD (10, 14). Nevertheless, further research is needed to elucidate the molecular targets associated with pharmacological effects through which these effects are exerted.

Network pharmacology, an emerging methodology in drug discovery and development, integrates laboratory and clinical investigations with data analysis to comprehensively elucidate the pharmacological effects of traditional Chinese medicines on a range of disorders (15, 16). It is a comprehensive methodology that combines conventional pharmacology, bioinformatics, chemoinformatics, and systems biology to construct networks of interactions between drugs, targets, and diseases, thereby facilitating the discovery of innovative therapeutic interventions (17). Concurrently, it has been extensively employed to investigate the multi-target effects linked to central nervous system disorders, including ischemic stroke, vascular cognitive impairment, and AD (16, 18).

This study aimed to investigate the therapeutic targets of LJF and elucidate its anti-AD mechanisms using network pharmacology, Gene Expression Omnibus (GEO) datasets, molecular docking and dynamics simulation. Furthermore, we analyzed putative targets linked to Aβ and tau pathology. This study successfully predicted six potential core targets, including mitogen-activated protein kinase 8 (MAPK8), catenin beta-1 (CTNNB1), nuclear factor NF-kappa-B p105 subunit (NFKB1), epidermal growth factor receptor (EGFR), apoptosis regulator Bcl-2 (BCL2), and nuclear factor erythroid 2-related factor 2 (NFE2L2), and six effective components, including lignan, β-carotene, β-sitosterol, hederagenin, berberine, and baicalein, involved in the therapeutic strategy for AD using LJF. Our study offers a comprehensive pharmacological rationale for utilizing LJF in treating AD. Furthermore, this study significantly contributes to the advancement and application of LJF, guiding future research endeavors in this field.

## 2 Materials and methods

### 2.1 Screening of bioactive constituents of LJF

The search term “*Lonicerae Japonicae Flos*” was employed to access the active constituents within the SymMap database (oral bioavailability ≥30%) (19), the BATMAN-TCM online bioinformatics analysis tool (score cutoff = 20, adjusted *p*-value <0.05) (20), the Traditional Chinese Medicine Systems Pharmacology Database and Analysis Platform (TCMSP) database (oral bioavailability ≥30%, drug-likeness ≥0.18) (17), and the Encyclopedia of Traditional Chinese Medicine (ETCM) database (drug-likeness weight ≥0.5) (21).

### 2.2 Screening of potential targets mapped by ingredients

The active components were screened for their targets using four databases, namely SymMap [false discovery rate (Benjamini–Hochberg) <0.05], BATMAN-TCM (score cutoff = 20, adjusted *p*-value <0.05), TCMSP (random forest score ≥0.7, support vector machine score ≥0.8), and ETCM (similarity value >0.8), with a filter for *Homo sapiens species*. The target names of the proteins under investigation were consistently standardized using the RCSB Protein Data Bank database.^1^ Duplicate targets were eliminated, and related targets were retained for subsequent analysis.

### 2.3 Screening of potential targets of AD

Four disease databases included the MalaCards database (22), the GeneCards database,^2^ the DisGeNET database (23), and the Online Mendelian Inheritance in Man (OMIM) database^3^ were utilized to screen potential targets related to AD using the keyword “Alzheimer’s disease.” To enhance the correlation with AD, we filtered targets with a relevance score ≥10 in the MalaCards database, score ≥10 in the GeneCards database, score ≥0.05 in the DisGeNET database, and selected all targets in the OMIN database. Redundant targets were removed, while relevant targets were preserved for subsequent analysis.

### 2.4 Acquisition of intersection targets

To obtain the relationship between AD-related and LJF-related targets, we conducted an intersection analysis of the respective target lists using an online interactive tool that facilitates comparison through Venn diagrams.^4^ We retained the intersection targets for subsequent analysis.

### 2.5 Function and pathway enrichment analyses

The Database for Annotation, Visualization and Integrated Discovery (DAVID)^5^ was utilized to distinguish and enrich the biological attributes, including biological processes, cellular components, molecular functions, and pathways. A false discovery rate (FDR) <0.05 significance threshold was established for enrichment analysis. The top 20 terms were selected for visualization according to their frequency count.

### 2.6 Analyses of protein–protein interaction network and putative core targets

Intersection targets were chosen and input into the STRING database^6^ to retrieve the protein interaction network with a confidence score of 0.4 or higher. The results were input into the Cytoscape 3.7.1 software to establish and analyze the interaction network (24). In order to identify potential core targets, the cytoHubba plugin was utilized to evaluate and prioritize nodes (proteins) based on network characteristics with seven scoring algorithms, including maximal clique centrality (MCC), density of maximum neighborhood component (DMNC), maximum neighborhood component (MNC), edge percolated component (EPC), degree, closeness, and betweenness (25). Ultimately, this study identified putative core targets that exhibited concordance across three or more topological algorithms.

### 2.7 Expression validation of putative core targets

The postulated core targets were validated differential expression in GEO datasets. To assess expression levels of putative core targets between AD cases and normal controls, we conducted differential expression analyses through the comprehensive AlzData database^7^ with data from the hippocampal datasets, including GSE28146 (expression data from early-stage AD), GSE29378 (expression data from late-stage AD), GSE36980 (expression data from post mortem AD brains), GSE48350 (expression data from post mortem AD brains), and GSE5281 (undistinguished stages). The data were shown as mean ± standard deviation, with a *p*-value of <0.05 indicating significant differences. Differentially expressed core targets were utilized for further validation.

### 2.8 Correlation analysis between core target genes and main causative genes of AD

To further validate the relativity with AD, correlation analysis was performed on putative core target genes and major causative genes, including amyloid precursor protein (APP), microtubule-associated protein tau (MAPT), and presenilin2 (PSEN2) through the gene expression profiling interactive analysis (GEPIA) (26). Putative core target genes were imported into the search words, and brain-hippocampus was selected from GTEx expression datasets. We selected genes that exhibited a significant association with major causative genes of AD for further validation.

### 2.9 Receiver operating characteristic analysis

To evaluate the predictive efficacy of core targets, we performed a ROC analysis, calculating the area under the receiver operating characteristic (ROC) curve (AUC) as a metric for their predictive capability. Therefore, we applied ROC curve analysis with hippocampus datasets, including GSE28146, GSE29378, GSE36980, GSE48350, and GSE5281, to evaluate these core targets’ predictive utility.

### 2.10 Identification of bioactive ingredients interacting with core targets

Four databases, including SymMap, BATMAN-TCM, TCMSP, and ETCM, were used to investigate the essential bioactive constituents of core targets. Duplicate ingredients were removed, while pertinent ingredients were retained. To elucidate the interaction between core targets and essential ingredients, we intersected ingredients of core targets mapped and LJF using an online tool to compare lists with Venn diagrams. Intersection ingredients with oral bioavailability (OB) ≥0.3, drug-likeness (DL) ≥0.18, and blood-brain barrier (BBB) ≥−0.3 were retained for subsequent analysis.

### 2.11 Molecular docking

The AutoDock Vina software evaluated the molecular binding affinity between bioactive constituents and their corresponding core targets. The two-dimensional molecular structures of bioactive compounds in LJF were acquired from the PubChem database.^8^ The crystallographic structures of core target proteins were acquired from the RCSB Protein Data Bank and SWISS-MODEL.^9^ PyMOL^10^ and AutoDock Vina were employed to structurally manipulate the proteins, involving eliminating water molecules and heteroatoms and incorporating charges and hydrogen atoms. The resulting profiles were then saved in the PDBQT format for binding studies. Subsequently, docking analyses were conducted utilizing AutoDock Vina, and the resulting docking conformations were visualized using PyMOL and Discovery Studio 2020 software. The results were presented through two-dimensional (2D) and three-dimensional (3D) graphical representations. Affinity was utilized to assess the binding efficacy of the primary active compounds with their respective targets. The affinity values of −4.25 kcal/mol, −5.0 kcal/mol, and −7.0 kcal/mol suggest varying degrees of binding strength between the compound and the target, with lower values indicating stronger binding and higher values indicating weaker binding interactions (24).

### 2.12 Molecular dynamics simulation

The molecular dynamics simulations of protein-component complexes derived from molecular docking were conducted using GROMACS (version 2020.6).^11^ The receptor proteins topology files were created utilizing the AMBER99SB force field, while the ligands component topology files were generated by applying the sobtop script with the AMBER force field. The system was neutralized with NaCl counterions. Before conducting the dynamics simulation, the complex underwent minimization for 10,000 steps and equilibration by executing canonical ensemble and constant-pressure, constant-temperature simulations for 100 picoseconds. The Coulomb force intercept and van der Waals radius intercept measured 1.4 nm. The system was subsequently equilibrated with the regular and isothermal isobaric system, followed by molecular dynamics simulations conducted for 10 nanoseconds under room temperature and pressure conditions. Ultimately, the root mean square deviation (RMSD) curves of protein-component complexes were analyzed to assess binding stability.

## 3 Results

### 3.1 Screening of potential targets of LJF against AD

**Figure 1:**
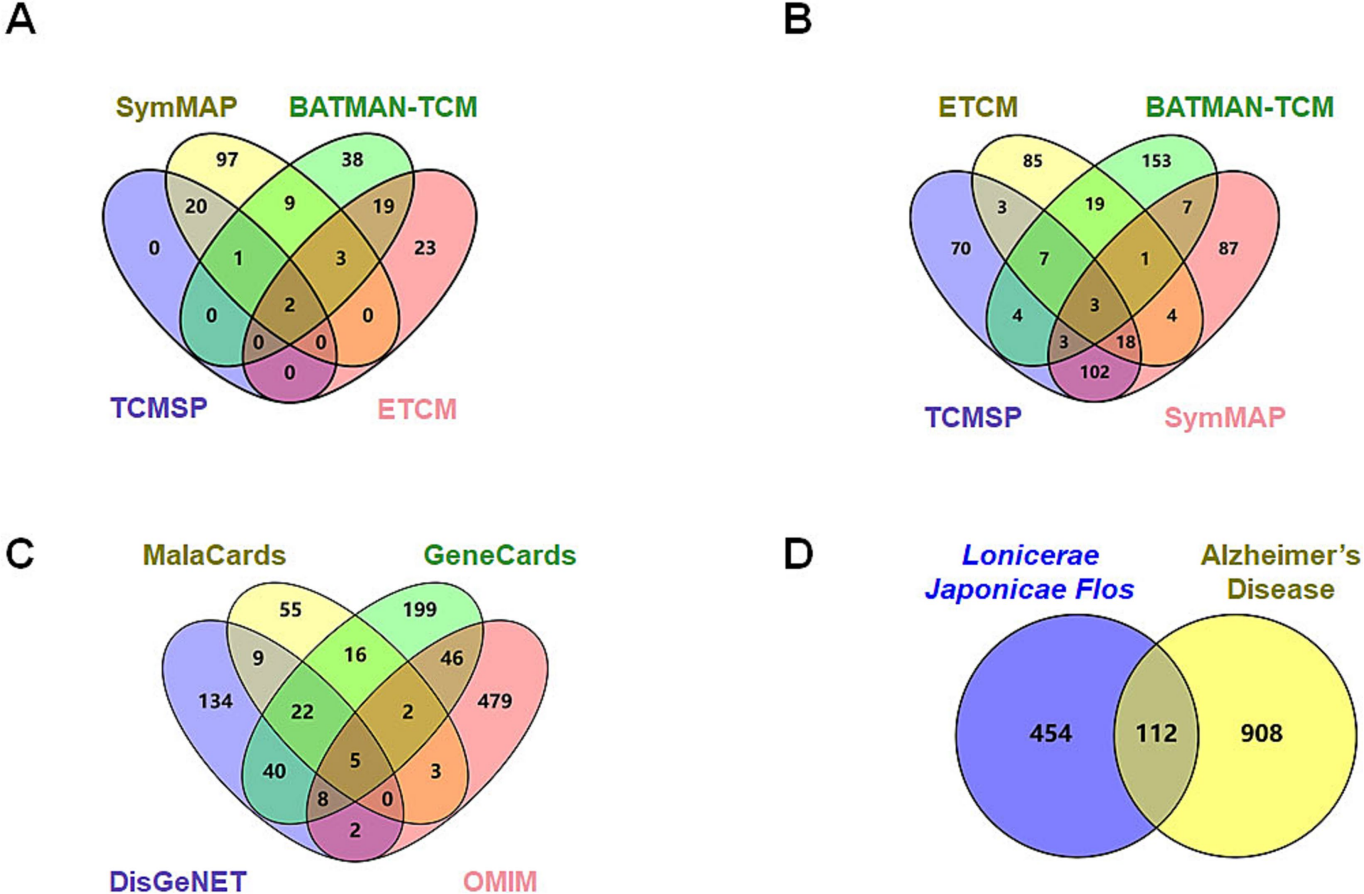
Screening of potential targets of *Lonicerae Japonicae Flos* in the treatment of AD. (A) Venn diagrams showing the numbers of bioactive compounds of *Lonicerae Japonicae Flos* in each database. The purple is TCMSP (23 ingredients), green is BATMAN-TCM (72 ingredients), red is ETCM (47 ingredients), and yellow is SymMap (132 ingredients). (B) Venn diagrams show the numbers of potential targets for ingredients in each database. The purple is TCMSP (210 potential targets), green is BATMAN-TCM (197 potential targets), red is SymMap (225 potential targets), and yellow is ETCM (140 potential targets). (C) Venn diagrams show the numbers of potential AD targets in each database. The purple is DisGeNET (220 potential targets), green is GeneCards (338 potential targets), red is OMIM (545 potential targets), and yellow is MalaCards (112 potential targets). (D) Venn diagram showing numbers of intersection targets. The purple is *Lonicerae Japonicae Flos* (566 potential targets), and the yellow is AD (1,020 potential targets).

Active constituents of LJF were identified through a comprehensive review of relevant literature from databases, including SymMap, BATMAN-TCM, TCMSP, and ETCM. A total of 132 active ingredients with an OB score ≥0.3 were identified from the SymMap database, 72 active ingredients with a score exceeding 20 from the BATMAN-TCM database, 23 active ingredients with an OB ≥0.3 and DL ≥0.18 from the TCMSP database, and 47 active constituents from the ETCM database were found to be effective (Figure 1A). Following the screening criteria, 212 species were identified after eliminating duplicates. The SymMap, BATMAN-TCM, TCMSP, and ETCM databases were utilized to identify potential targets of efficacious active compounds. The UniProt database was utilized to standardize the official names of all retrieved proteins. All duplication proteins were removed after merging to screen 566 potential targets (Figure 1B). MalaCards, GeneCards, DisGeNET, and OMIM databases were queried with the subject heading “Alzheimer’s disease” to screen potential targets. The MalaCards database yielded 112 related targets with a score greater than 10, the GeneCards database identified 338 related targets with gifts exceeding 30 and relevance surpassing 25, the DisGeNET database revealed 220 related targets with a score exceeding 0.1, and the OMIM database provided information on 545 related targets (Figure 1C). Four sources of targets were merged and duplication was removed to yield 1,020 AD-related targets. A total of 112 intersection targets were identified between LJF and AD by constructing Venn diagrams, as shown in Figure 1D.

### 3.2 GO and KEGG enrichment analyses of intersection targets

**Figure 2:**
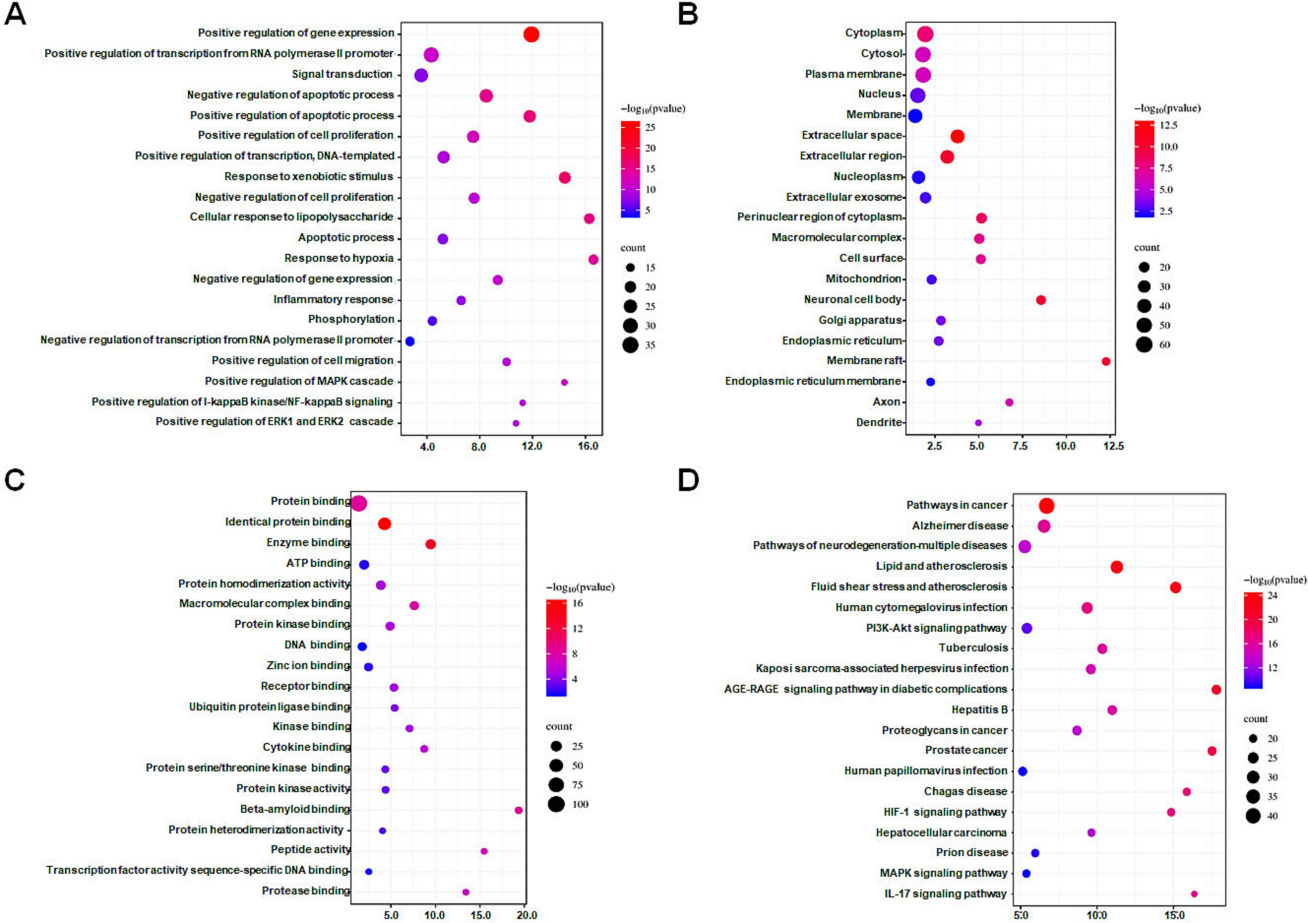
Bubble diagrams of GO and KEGG enrichment analysis of intersection targets. (A) Biological process (BP) enrichment analysis showing the top 20 terms. (B) Cell composition (CC) enrichment analysis showing the top 20 terms. (C) Molecular function (MF) enrichment analysis showing the top 20 terms. (D) KEGG enrichment analysis showing the top 20 terms. The *X*-axis label represents the values of the count, and the *Y*-axis label represents the terms. A false discovery rate <0.05.

The DAVID database was utilized to perform GO and KEGG enrichment analyses on 112 intersection targets. With FDR <0.05 as screening conditions, 923 GO entries (biological process 694 entries, cell composition 103 entries, and molecular function 126 entries) and 175 KEGG pathways entries were acquired. According to counts and the FDR value, the top 20 items in the three biological attributes were selected to draw bubble diagrams, as shown in Figures 2A–C. In terms of biological processes, intersection targets are primarily involved in positive regulation of gene expression (GO: 0010628), positive regulation of transcription from RNA polymerase II promoter (GO: 0045944), signal transduction (GO: 0007165), negative regulation of apoptotic process (GO: 0043066), positive regulation of apoptotic process (GO: 0043065), etc. In terms of cellular components, intersection targets are primarily involved in the cytoplasm (GO: 0005737), cytosol (GO: 0005829), plasma membrane (GO: 0005886), nucleus (GO: 0005634), membrane (GO: 0016020), etc. In terms of molecular functions, intersection targets are primarily involved in protein binding (GO: 0005515), identical protein binding (GO: 0042802), enzyme binding (GO: 0019899), ATP binding (GO: 0005524), protein homodimerization activity (GO: 0042803), etc. For the KEGG pathway enrichment, the re-screening condition is that the number of enriched targets is ≥20. The bubble chart of the top 20 pathways is ranked according to the counts, and FDR value is shown in Figure 2D. Intersection targets are primarily involved in pathways in cancer (hsa05200), Alzheimer disease (hsa05010), pathways of neurodegeneration—multiple diseases (hsa05022), lipid and atherosclerosis (hsa05417), fluid shear stress and atherosclerosis (hsa05418), etc.

### 3.3 PPI network construction and core targets analysis

**Figure 3:**
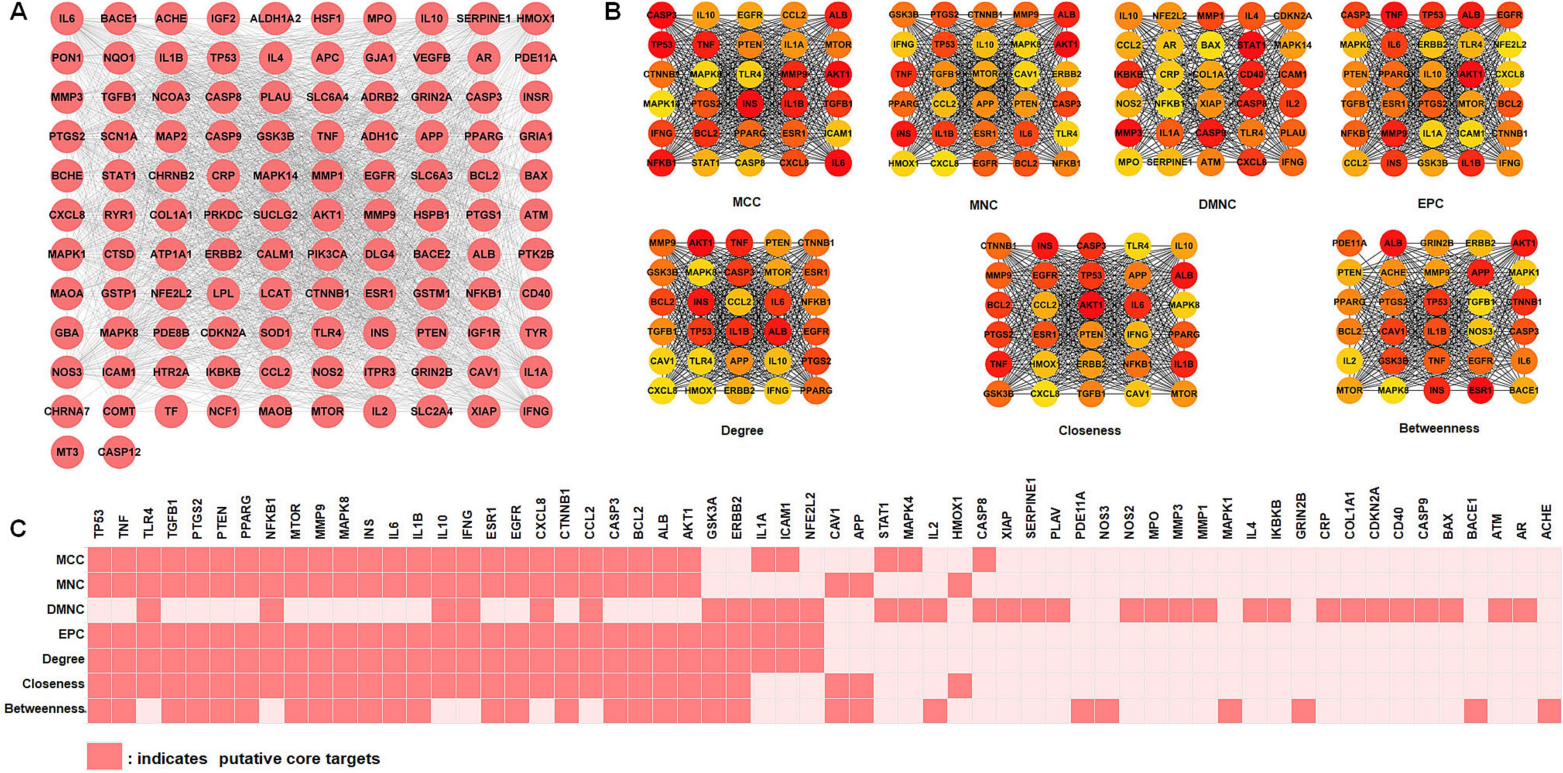
Identification of core targets via protein–protein interaction (PPI) analysis. (A) PPI network analysis of 112 intersection targets. (B) The top 30 hub targets were chosen from the PPI network using the cytoHubba plugin (MCC, MNC, DMNC, EPC, degree, closeness, and betweenness). (C) Distribution of top 30 hub targets identified by topological analysis methods. MCC, maximal clique centrality; DMNC, density of maximum neighborhood component; MNC, maximum neighborhood component; EPC, edge percolated component.

To improve comprehension of targets interactions, the STRING online tool was utilized to analyze the protein–protein interaction (PPI) network involving intersection targets. The Cytoscape software presented 112 nodes (targets) and 2,069 edges (interactions), as shown in Figure 3A. We used the MCC, MNC, DMNC, EPC, degree, closeness, and betweenness algorithms in the cytoHubba plug-in of the Cytoscape software to select potential core targets and the top 30 targets of each algorithm for intersections, as shown in Figure 3B. As shown in Figure 3C, 32 putative core targets (TP53, TNF, TLR4, TGFB1, PTGS2, PTEN, PPARG, NFKB1, MTOR, MMP9, MAPK8, INS, IL6, IL1B, IL10, IFNG, ESR1, EGFR, CXCL8, CTNNB1, CCL2, CASP3, BCL2, ALB, AKT1, GSK3A, ERBB2, IL1A, ICAM1, NFE2L2, CAV1, and APP) shared more than three topological methods were obtained.

### 3.4 Differential expression evaluation of putative core targets based on GEO datasets

**Figure 4:**
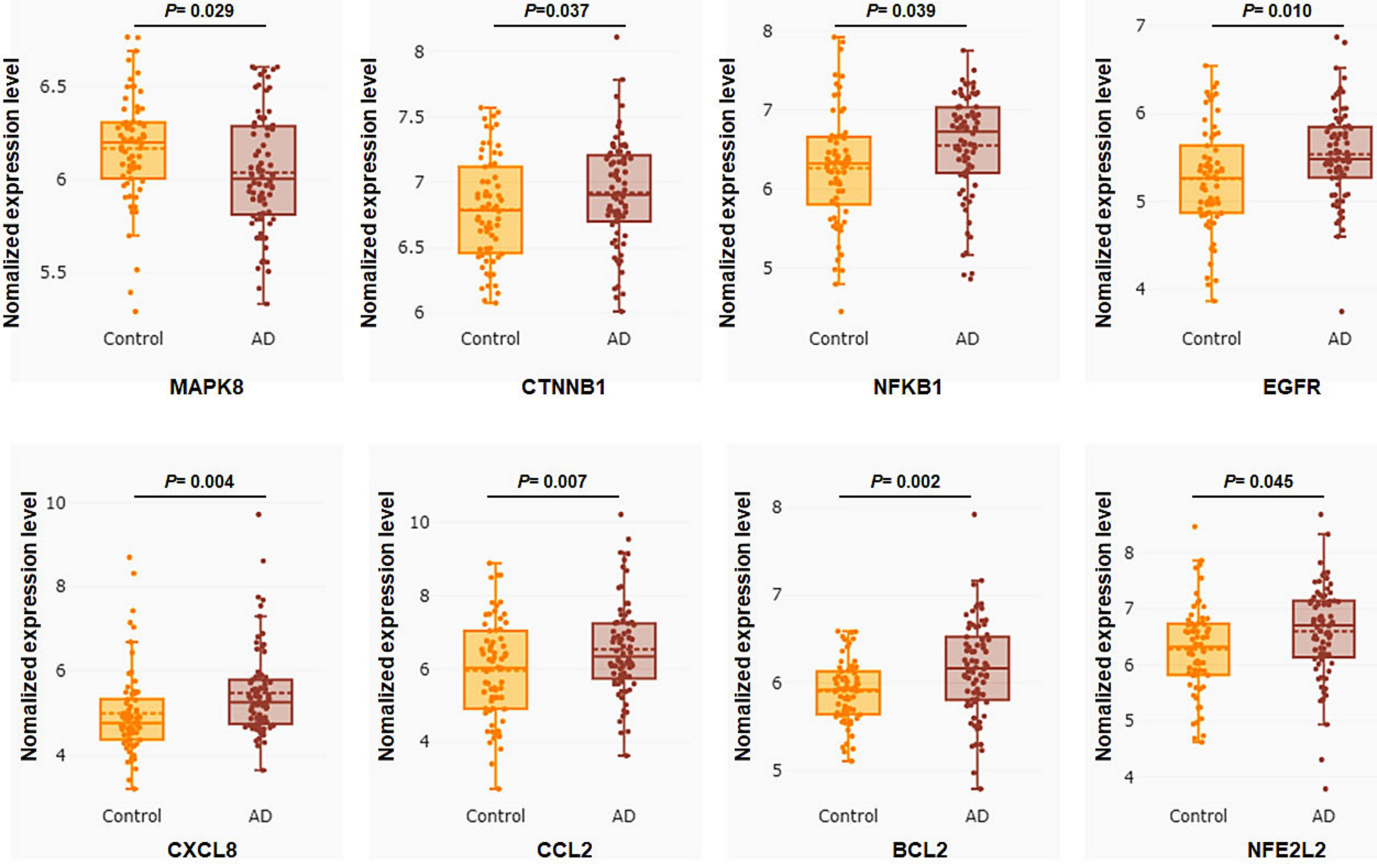
Core targets differential expression validation between control and AD groups of GEO datasets. Target gene expression data were compared to the control and AD groups in the human hippocampus in the datasets GSE28146, GSE29378, GSE36980, GSE48350, and GSE5281. Control group (*N* = 66) and AD group (*N* = 74). Data are displayed as means ± standard errors. *p* < 0.05 was deemed statistically significant.

To verify the clinical implications of 32 putative core targets, the brain transcriptome datasets of GSE28146, GSE29378, GSE36980, GSE48350, and GSE5281, which examined the expression of human hippocampus in normal control (*N* = 76) and AD (*N* = 74) patients, were used to assess the expression changes in brain hippocampus tissue of AD patients. The result showed that MAPK8 significantly down-regulated, CTNNB1, NFKB1, EGFR, CXCL8, CCL2, BCL2, and NFE2L2 were significantly up-regulated in AD patients compared to controls (Figure 4). However, there was no significant difference in TP53, TNF, TLR4, TGFB1, PTGS2, PTEN, PPARG, MTOR, MMP9, INS, IL6, IL1B, IL10, IFNG, ESR1, CASP3, ALB, AKT1, GSK3A, ERBB2, IL1A, ICAM1, CAV1, and APP levels between control and AD groups in the hippocampus (data not shown). These results showed that only core targets of MAPK8, CTNNB1, NFKB1, EGFR, CXCL8, CCL2, BCL2, and NFE2L2 might play a significant role in AD progression.

### 3.5 Correlation analysis between putative core targets and main causative genes of AD

**Figure 5:**
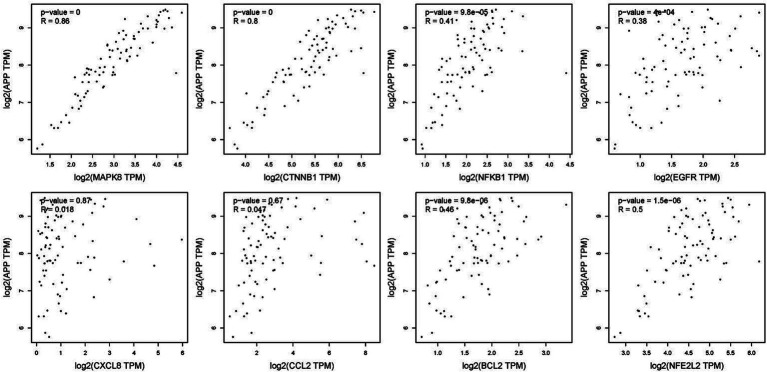
Verification of dependencies between hub targets and APP protein.

**Figure 6:**
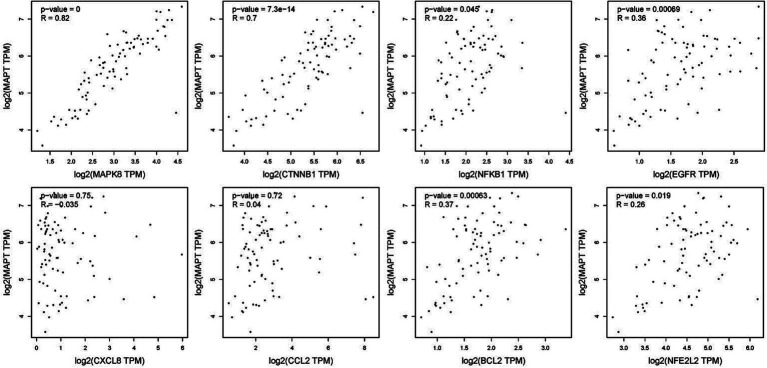
Verification of dependencies between hub targets and tau protein.

**Figure 7:**
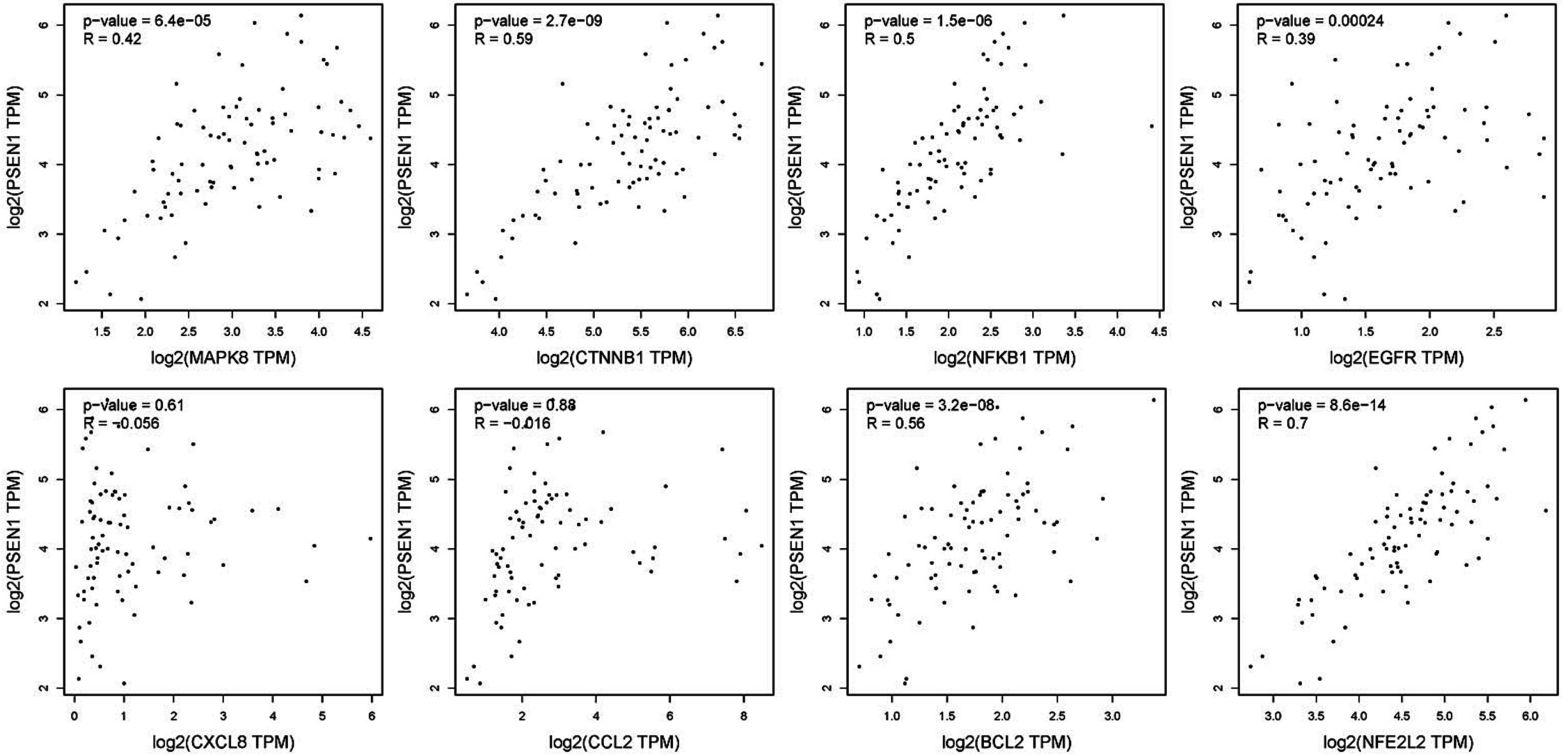
Verification of dependencies between hub targets and PSEN1 protein.

To verify the correlation between core targets screened by differential expression and the main causative genes APP, MAPT and PSEN2, correlation analysis was performed using GEPIA. The results displayed that only core targets of MAPK8, CTNNB1, NFKB1, EGFR, BCL2, and NFE2L2 were significantly related to APP, MAPT, and PSEN2 (Figures 5–7). It is well-established that six core targets of MAPK8, CTNNB1, NFKB1, EGFR, BCL2, and NFE2L2 were the most prominent factors in treating LJF against AD.

### 3.6 ROC curves analysis of core targets

**Figure 8:**
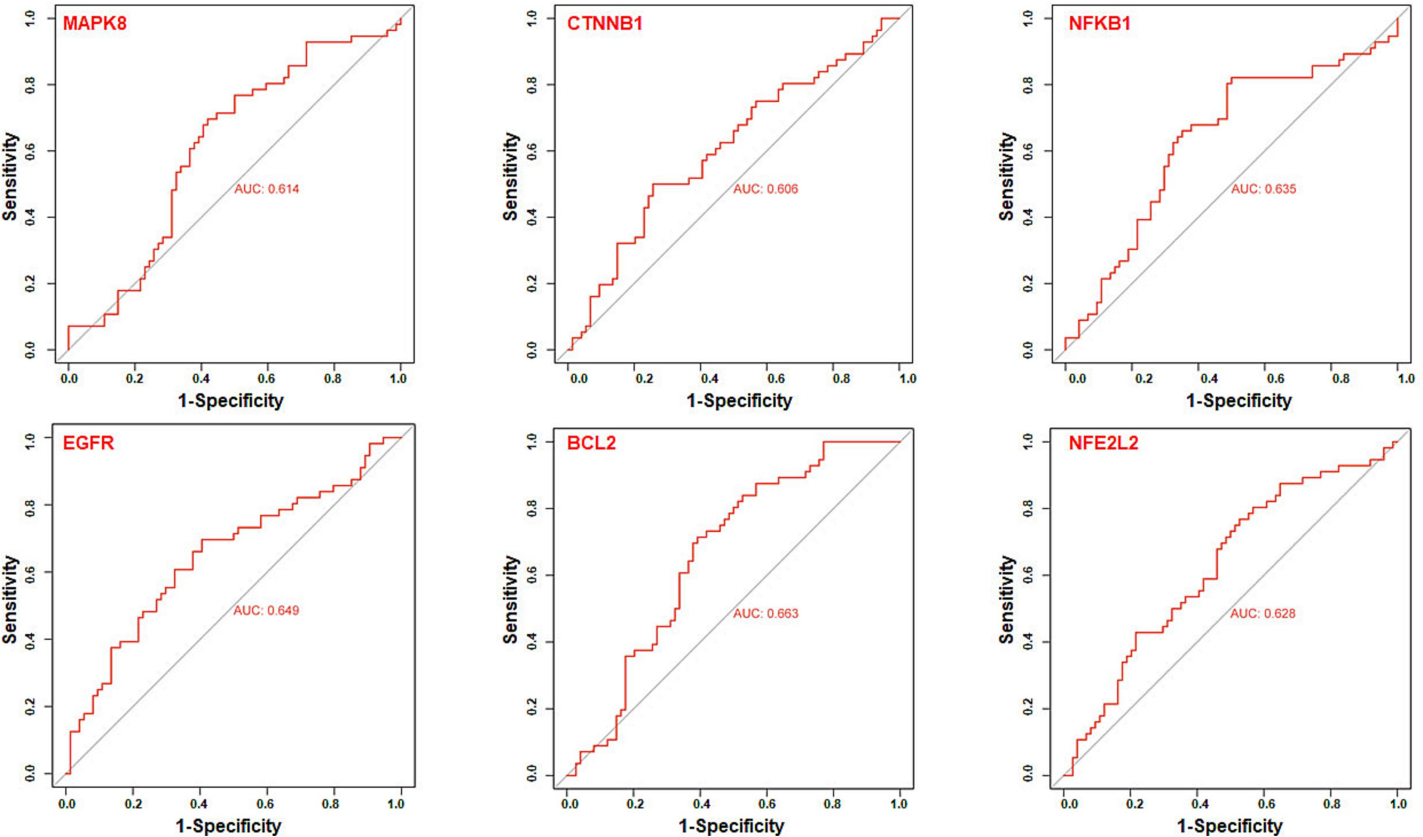
Receiver operating characteristic curve analysis of core targets. The receiver operating characteristic curve evaluated the excellent clinical predictive utility of core targets based on GEO datasets of the human hippocampus (GSE28146, GSE29378, GSE36980, GSE48350, and GSE5281). Control group (*N* = 66) and AD group (*N* = 74). *p* < 0.05 was deemed statistically significant.

We further utilize gene expression datasets of the human hippocampus (GSE28146, GSE29378, GSE36980, GSE48350, and GSE5281) to establish ROC curves to validate six core targets of MAPK8, CTNNB1, NFKB1, EGFR, BCL2, and NFE2L2 related to AD in diagnosis, as shown in Figure 8. The area under the ROC curve, commonly denoted as AUC, serves as a metric for evaluating the overall effectiveness of a predictive model. As the AUC score approaches 1, the diagnostic performance is better. The results showed that core targets of MAPK8 (AUC = 0.614), CTNNB1 (AUC = 0.606), NFKB1 (AUC = 0.635), EGFR (AUC = 0.649), BCL2 (AUC = 0.663), and NFE2L2 (AUC = 0.628) have good predictive ability. These results indicated that six core targets of MAPK8, CTNNB1, NFKB1, EGFR, BCL2, and NFE2L2 are associated with AD, indicating good specificity and sensitivity for diagnosing patients, as shown in Table 1. Accordingly, we speculated that MAPK8, CTNNB1, NFKB1, EGFR, BCL2, and NFE2L2 mediated the therapeutic effects of LJF against AD. But still we need to admit that the ROC analysis results for the predictive ability of the identified core targets show moderate values of AUC, it is necessary to further validate in the external and cross datasets.

**Table 1:** The core targets information associated with Alzheimer’s disease.

Gene	UniProt	Protein name	Alteration	Relationship to AD	References
MAPK3	Q16644	MAP kinase-activated protein kinase 3	Up-regulation	Prevent AD	(45, 46)
CTNNB1	P35222	Catenin beta-1	Down-regulation	Prevent AD	(35)
NFKB1	P19838	Nuclear factor NF-kappa-B p105 subunit	Activated NFKB1	Promote the development of AD	(39)
EGFR	P00533	Epidermal growth factor receptor	Up-regulation	Promote the development of AD	(31)
BCL2	P10415	Apoptosis regulator Bcl-2	up-regulation	Prevent AD	(33)
NFE2L2	Q16236	Nuclear factor erythroid 2-related factor 2	Activated NFE2L2	Prevent AD	(48)

### 3.7 Bioactive ingredients of LJF mapped by core targets

**Figure 9:**
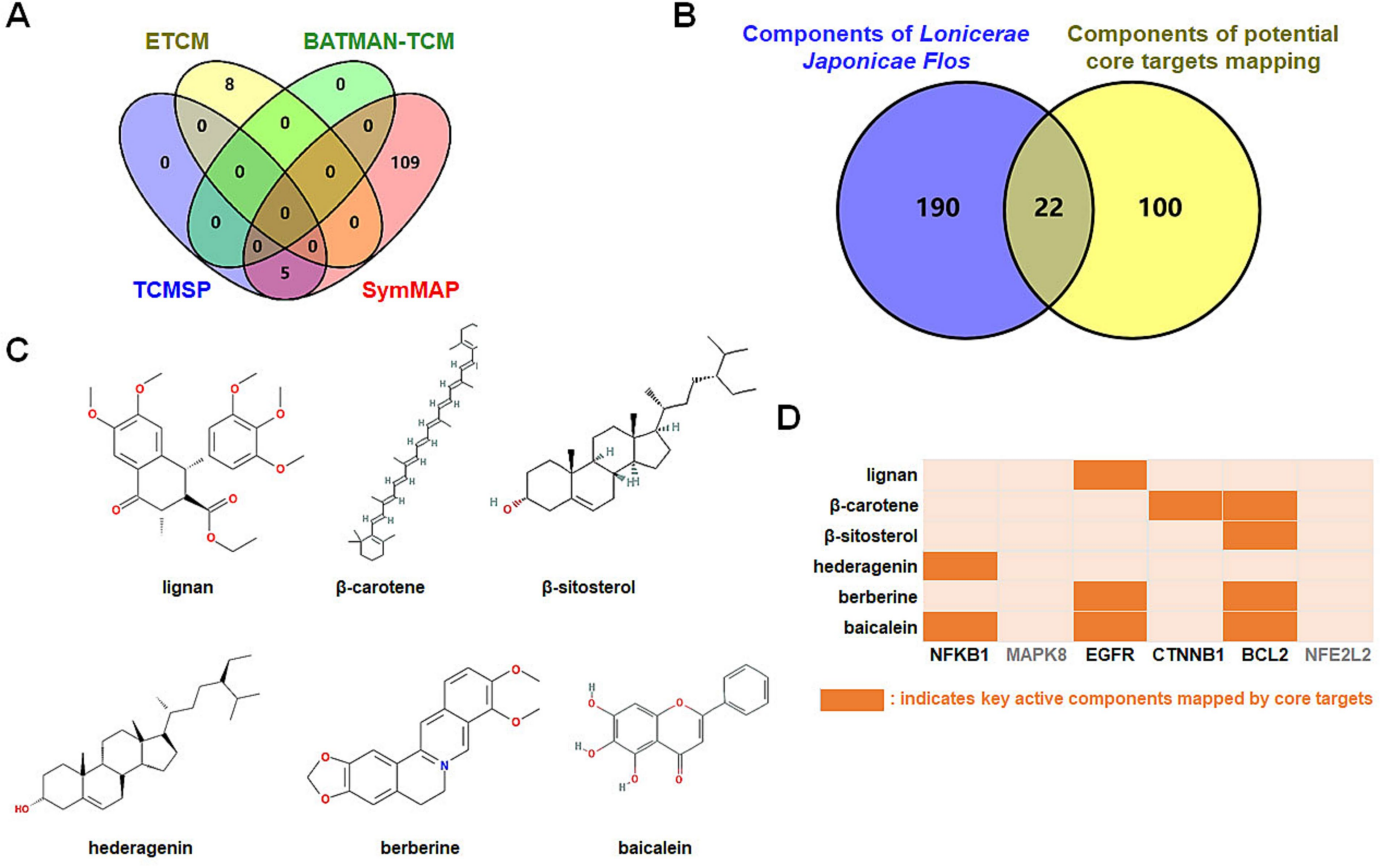
Identification of bioactive ingredients of *Lonicerae Japonicae Flos*. (A) Venn diagrams show the numbers of potential bioactive ingredients mapped by hub targets in each database. The purple is TCMSP (5 key ingredients), green is BATMAN-TCM (0 key ingredients), red is SymMap (114 key ingredients), and yellow is ETCM (8 key ingredients). (B) Venn diagram showing numbers of *Lonicerae Japonicae Flos* intersection ingredients and hub targets. (C) Chemical structure formulas of crucial active ingredients. (D) Mapping between critical components and core targets.

Which chemicals in LJF can target the identified core targets? To address this question, SymMap, BATMAN-TCM, TCMSP, and ETCM databases were utilized to screen for potential effective compounds of core targets of MAPK8, CTNNB1, NFKB1, EGFR, BCL2, and NFE2L2 (Figure 9A). In order to clarify the target-active compounds mapping relationship, we intersected the 212 LJF components and 122 core target components with an online interactive tool for comparing lists with Venn’s diagrams (Figure 9B). A total of 22 putative components were obtained (Table 2). After filtration with OB ≥0.3, DL ≥0.18, and BBB≥−0.3, only six effective active ingredients, including lignan (OB = 43.32, BBB = −0.16, DL = 0.65), β-carotene (OB = 37.18, BBB = 1.52, DL = 0.58), β-sitosterol (OB = 36.91, BBB = 0.99, DL = 0.75), hederagenin (OB = 36.91, BBB = 0.96, DL = 0.75), berberine (OB = 36.86, BBB = 0.57, DL = 0.78), and baicalein (OB = 33.52, BBB = −0.05, DL = 0.21), were screened, and the structures of which were shown in Figure 9C. Accordingly, we speculated that lignan, β-carotene, β-sitosterol, hederagenin, berberine, and baicalein mediated the preventive and therapeutic properties of LJF against AD. Intriguingly, the core targets of MAPK8 and NFE2L2 were not mapped by any effective active ingredients (Figure 9D).

**Table 2:** Twenty-two putative active compounds mapped by core targets.

MOL ID	Molecule name	MW	OB (%)	BBB	DL	TCMSP	SymMap	BATMAN-TCM	ETCM
TCMID13295	Macranthoside A	913.10	None	None	None	−	−	+	+
TCMID15511	New triterpennoid glycoside	1399.52	None	None	None	−	−	+	+
TCMID13296	Macranthoside B	1075.24	None	None	None	−	−	−	+
TCMID11118	Ioniceroside C	1075.24	None	None	None	−	−	−	+
MOL001924	Paeoniflorin	480.51	53.87	−1.86	0.79	−	+	−	−
MOL001454	Berberine^a^	336.39	36.86	0.57	0.78	−	+	−	−
MOL000359	β-sitosterol^a^	414.79	36.91	0.99	0.75	+	+	−	−
MOL000296	Hederagenin^a^	414.79	36.91	0.96	0.75	−	+	+	+
MOL002776	Baicalin	446.39	40.12	−1.74	0.75	−	+	−	−
MOL002695	Lignan^a^	458.55	43.32	−0.16	0.65	−	+	−	−
MOL000357	Sitogluside	576.95	20.63	−0.93	0.62	−	−	−	+
MOL002773	β-carotene^a^	536.96	37.18	1.52	0.58	+	+	−	−
MOL000098	Quercetin	302.25	46.43	−0.77	0.28	+	+	−	−
MOL000006	Luteolin	286.25	36.16	−0.84	0.25	+	+	+	+
MOL000422	Kaempferol	286.25	41.88	−0.55	0.24	+	+	−	−
MOL002714	Baicalein^a^	270.25	33.52	−0.05	0.21	−	+	−	−
MOL000414	Caffeate	180.17	54.97	0.11	0.05	−	+	−	−
MOL000254	Eugenol	164.22	56.24	1.32	0.04	−	+	+	−
MOL002042	Thymol	150.24	41.47	1.68	0.03	−	+	−	+
MOL000700	Nerol	154.28	35.66	1.14	0.02	−	+	+	−
MOL000745	Macranthoidin A	1237.55	4.06	−4.94	0.01	−	−	+	+
MOL003042	Macranthoidin B	1399.71	6.69	−6.98	0.01	−	−	+	+

MW, molecular weight; OB, oral bioavailability; BBB, blood-brain barrier; DL, drug-likeness.

a Indicates effective compounds.

### 3.8 Molecular docking verification

**Figure 10:**
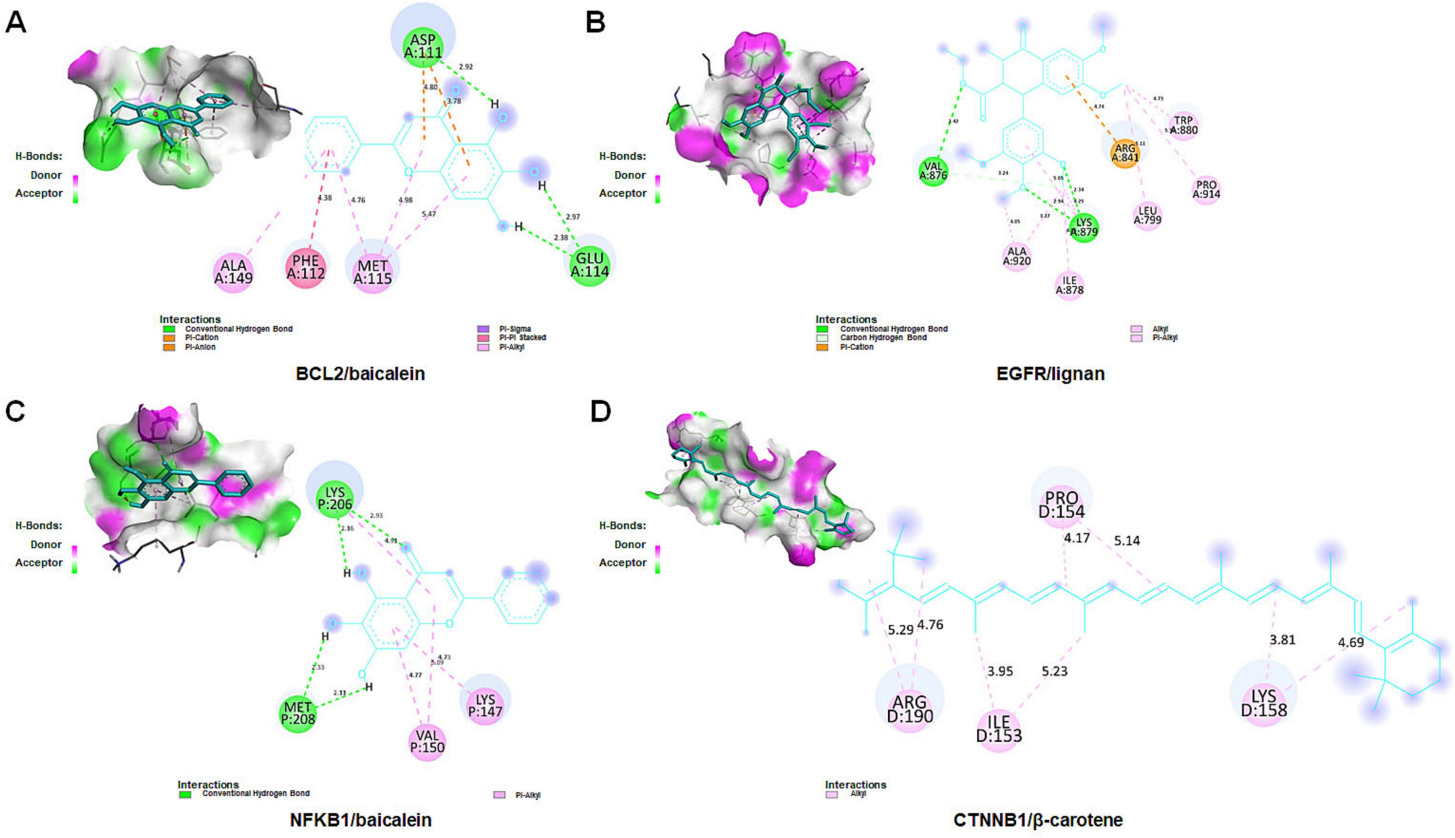
Molecular docking for crucial ingredients and hub targets. Molecular docking results for the BCL2/baicalein (A), EGFR/lignan (B), NFKB1/baicalein (C), and CTNNB1/β-carotene (D) complexes (3D and 2D).

To further validate the binding affinity of effective components (lignan, β-carotene, β-sitosterol, hederagenin, berberine, and baicalein) with core targets (CTNNB1, NFKB1, EGFR, and BCL2), molecular docking analysis was conducted utilizing AutoDock Vina, followed by visualization of the results using a visualization tool. The lower binding energy observed suggests a more favorable docking result. The binding energy (kcal/mol) and amino acid residues associated with the interaction between active ingredients and core targets were displayed in Table 3. BCL2 exhibited binding affinities of −8.1, −7.57, −7.14, and −6.13 kcal/mol towards berberine, β-sitosterol, β-carotene, and baicalein, respectively. EGFR demonstrated binding affinities of −6.59, −6.23, and −4.13 kcal/mol towards baicalein, berberine, and lignan, respectively. NFKB1 displayed binding affinities of −5.43 and −5.32 kcal/mol towards hederagenin and berberine, respectively. CTNNB1 exhibited a binding affinity of −5.85 kcal/mol towards β-carotene. Overall, lignan, β-carotene, β-sitosterol, hederagenin, berberine, and baicalein exhibit binding solid affinity towards their targets. The conformation with the lowest absolute binding energy values for each target was visualized using PyMoL and Discovery Studio 2020 software, as shown in Figure 10. Generally, lower free energy indicates a more stable structure. If a higher free energy conformation has been demonstrated to be effective binding for a ligand-receptor complex, representative conformations of lower free energies are not shown.

**Table 3:** Molecular docking between active compounds and core targets.

Protein	UniProt ID	Ligand	MOL ID	Binding energy (kcal/mol)	Amino acid residues
BCL2(6O0M)	P10415	Berberine	MOL001454	−8.1	Asp111, Phe112, Glu114, Met115, Val156
BCL2(6O0M)	P10415	β-sitosterol	MOL000358	−7.57	Phe112, Met115, Leu137
BCL2(6O0M)	P10415	β-carotene	MOL002773	−7.14	Leu104, Tyr108, Phe112, Met115, Leu119, Val133, Leu137, Ala149
BCL2(6O0M)	P10415	Baicalein	MOL002714	−6.13^a^	Asp111, Phe112, Glu114, Met115, Ala149
EGFR(8A27)	P00533	Baicalein	MOL002714	−6.59	Lys745, Met766, Cys775, Leu777, Leu788, Asp855, Phe856, Leu858
EGFR(8A27)	P00533	Berberine	MOL001454	−6.23	Lys806, Asp807, Phe910
EGFR(8A27)	P00533	Lignan	MOL002695	−4.13^a^	Leu799, Trp880, Arg841, Val876, Ile878, Lys879, Pro941, Ala920
NFKB1(1SVC)	P19838	Hederagenin	MOL000296	−5.43	His108, Gly169, His173, Pro174
NFKB1(1SVC)	P19838	Baicalein	MOL002714	−5.32^a^	Lys147, Val150, Lys206, Met208
CTNNB1(3SLA)	P35222	β-carotene	MOL002773	−5.85^a^	Ile153, Pro154, Lys158, Arg190

a The molecular docking mode of the lowest absolute values of binding energy of every target is shown in figures.

### 3.9 Molecular dynamics simulation

**Figure 11:**
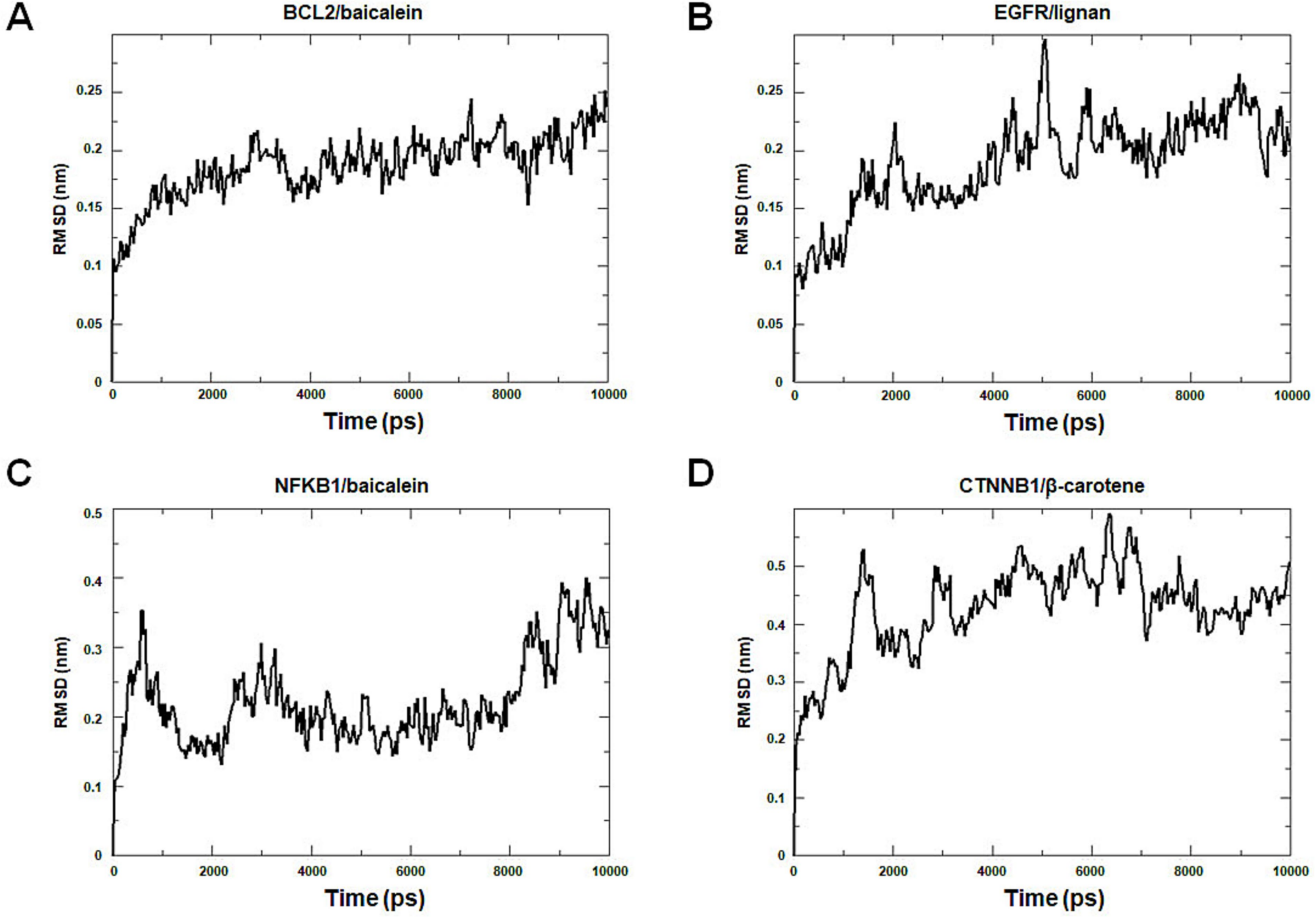
Molecular dynamics simulation analysis. The BCL2/baicalein (A), EGFR/lignan (B), NFKB1/baicalein (C), and CTNNB1/β-carotene (D) complexes root mean square deviation (RMSD) plot during molecular dynamics simulations. The *X*-axis represents the time (ps), and the *Y*-axis represents the RMSD (nm).

Molecular dynamics simulation offers more valuable insights into the stability of target-compound complexes. Molecular dynamics simulations were performed to assess binding affinities of BCL2/baicalein, EGFR/lignan, NFKB1/baicalein, and CTNNB1/β-carotene complexes. Generally, RMSD analysis is crucial for assessing the stability of targets and compounds, and lower RMSD values suggest more excellent stability in the target-compound complex. Figure 11 illustrates the fluctuation and stabilization of RMSD values for various protein-ligand complexes over time. Specifically, the RMSD of the BCL2/baicalein complex stabilized after 1,500 ps, while the EGFR/lignan complex reached stability at 5,000 ps. The NFKB1/baicalein complex showed stabilization between 4,000–8,000 ps, and the CTNNB1/β-carotene complex exhibited fluctuation before stabilizing at 7,000 ps. The RMSD values suggest that the active pockets of small molecules and proteins are stable. These findings indicate that the protein’s shape remains stable when the small molecule ligand binds to it, suggesting a strong interaction. These findings provide strong evidence in support of the accuracy of the molecular docking results.

### 3.10 Identification of new targets interacting with active ingredients

**Figure 12:**
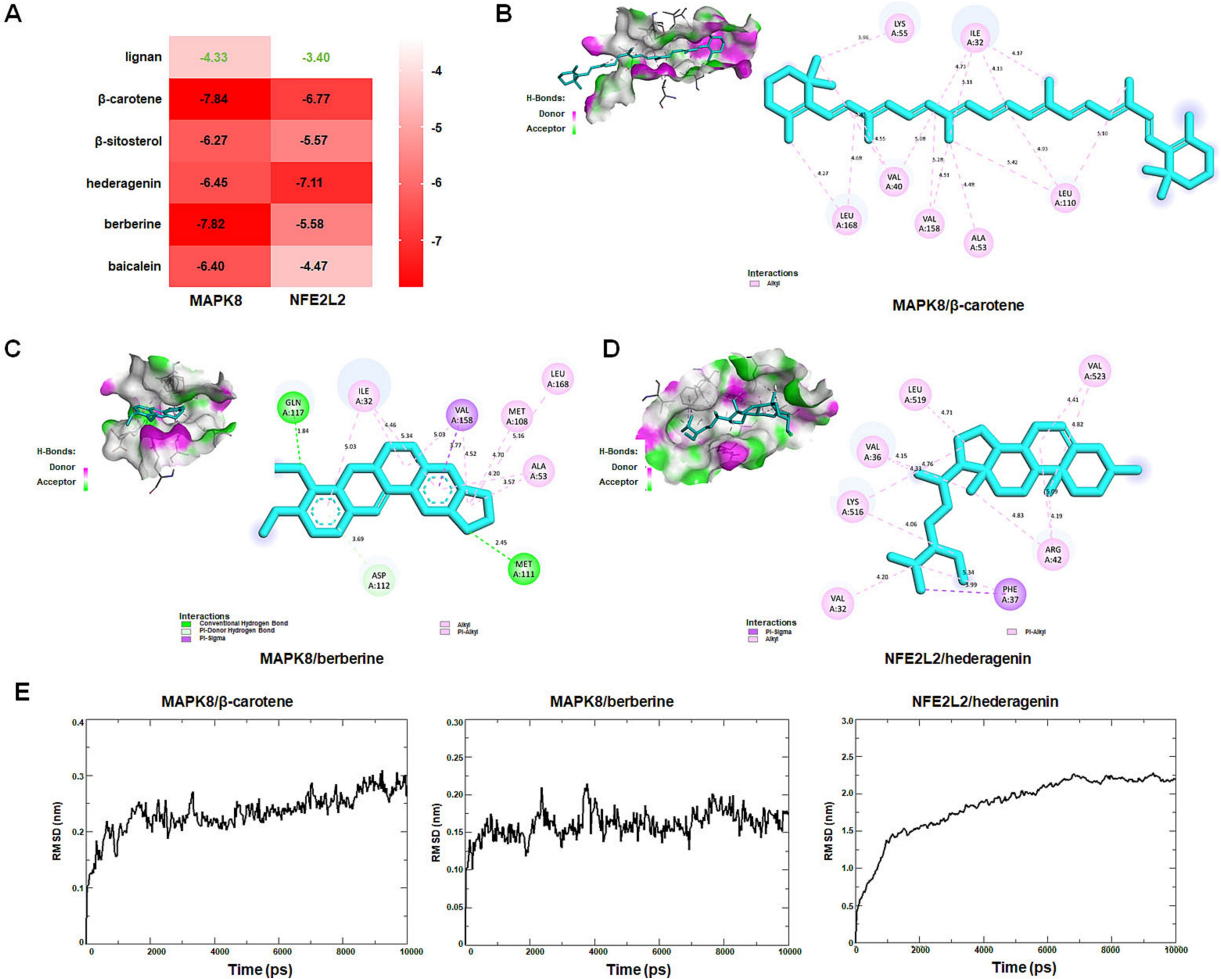
Identification of new targets by molecular docking and dynamics simulation. (A) The heat map shows the binding free energy (kcal/mol) of molecular docking. (B) Interactions between MAPK8 and β-carotene. (C) Interactions between MAPK8 and berberine. (D) Interactions between NFE2L2 and hederagenin. (E) MAPK8/β-carotene, MAPK8/berberine, and NFE2L2/hederagenin complexes root mean square deviation (RMSD) plot during molecular dynamics simulations. The *X*-axis represents the time (ps), and the *Y*-axis represents the RMSD (nm).

Molecular docking was performed to assess interactions between core targets (MAPK8 and NFE2L2) and active compounds, including lignan, β-carotene, β-sitosterol, hederagenin, berberine, and baicalein. The docking simulations showed strong binding affinities, with free energies ranging from −4.33 to −7.84 kcal/mol for MAPK8 and −3.40 to −7.11 kcal/mol for NFE2L2 (Figure 12A). The interactions between compounds and targets, characterized by binding energy scores <−7 kcal/mol (strong binding), were further examined using PyMoL and Discovery Studio 2020 software visualization techniques. Binding affinities were attributed to conventional hydrogen bond interactions with Met-111 and Gln-117 residues, Pi-donor hydrogen bond interactions with Asp-112 residue, Pi-Sigma interaction with Val-158 residue, as well as Pi-alky/alky interactions with Ile-32, Val-40, Ala-53, Lys-55, Met-108, Leu-110, Val-158, and Leu-168 residues of MAPK8 (Figures 12B,C). Binding affinities were attributed to Pi-sigma interaction with Phe-37 residue, Pi-alky/alky interactions with Val-32, Val-36, Arg-42, Lys-516, Leu-519, and Val-523 residues of NFE2L2 (Figure 12D). Molecular dynamics simulation provided insight into that the RMSD curves of the MAPK8/β-carotene complex stabilized after 1,500 ps, the RMSD curves of the MAPK8/berberine complex stabilized after 1,000 ps, and the RMSD curves of NFE2L2/hederagenin complex stabilized after 7,000 ps (Figure 12E). The findings of this study suggest that β-carotene and berberine exhibit significant binding affinity with MAPK8, while hederagenin demonstrates a binding solid affinity with NFE2L.

## 4 Discussion

AD is a persistent degenerative central nervous system disorder, distinguished by the accumulation of Aβ plaques, neuroinflammation, and oxidative stress (27). Individuals diagnosed with AD encounter challenges in communication, verbal expression, and cognitive processing, often accompanied by the gradual deterioration of long-term memory (4). Due to the intricate nature of its pathophysiological mechanism, AD has been a significant health concern. Therefore, the management of AD has become a prominent area of research. Currently, pharmaceutical interventions utilized for AD primarily consist of single-target medications that offer limited symptomatic relief and are associated with various adverse effects (5). Consequently, there is growing interest in developing multi-target drugs as a novel research focus. Traditional Chinese medicine has been a significant resource for medicinal treatments for centuries, employing systematic multi-target/multi-component strategies. Specifically, certain herbs within TCM have demonstrated pharmacological properties pertinent to AD (8, 28).

LJF refers to the dried bud or flower at the initial blooming stage of the plant. LJF is traditionally used for its medicinal properties, including clearing heat, detoxification, and dispersing wind heat. Chemical analysis has identified various components in LJF, such as essential oils, organic acids, flavonoids, iridoids, saponins, and other compounds (11). LJF demonstrates various effects, including anti-inflammatory, antioxidant, hypotensive, hypolipidemic, anti-thrombotic, and immunomodulatory properties (10). Prior research has indicated that LJF can inhibit the deposition of Aβ42 and decrease its neurotoxic effects on SH-SY5Y cells, thus indicating potential therapeutic implications for AD (14). Thus, a network pharmacology approach was employed with GEO datasets, molecular docking, and dynamics simulation to elucidate potential targets and compounds of LJF in the treatment of AD. Effective bioactive constituents and constituents-related targets were screened from SymMap, BATMAN-TCM, TCMSP, and ETCM databases. AD-related targets were identified using MalaCards, GeneCards, DisGeNET, and OMIM databases based on the scores assigned by the databases. Gene expression data sets of clinical AD patients were obtained from the GEO database to verify core target expression. The further validation of core targets entailed performing correlation analyses between these targets and the major causative genes associated with AD, as well as employing ROC curve modeling. The accuracy of the predictive results was validated through molecular docking and molecular dynamics simulations.

Through the analysis of the PPI network of intersection targets, we identified 32 putative core targets of LJF against AD. Gene expression analysis showed that the expression of MAPK8, CTNNB1, NFKB1, EGFR, CXCL8, CCL2, BCL2, and NFE2L2 increased or decreased in the hippocampus of patients with AD. To validate the correlation between core targets and AD, we performed the correlation analysis with major causative genes of AD and the ROC curve analysis. Correlation analysis showed that except for CXCL8 and CCL2, the other six targets (MAPK8, CTNNB1, NFKB1, EGFR, BCL2, and NFE2L2) were significantly positively linked to both Aβ and tau pathology of AD. ROC curve analysis showed that core targets of MAPK8, CTNNB1, NFKB1, EGFR, BCL2, and NFE2L2 have good predictive power, specificity, and sensitivity for diagnosing patients with AD. These results showed that core targets of MAPK8, CTNNB1, NFKB1, EGFR, BCL2, and NFE2L2 were involved in the treatment of LJF against AD.

Network pharmacology was utilized to identify bioactive ingredients related to core targets and investigate the underlying mechanism of LJF against AD. After filtration with OB ≥0.3, DL ≥0.18, and BBB ≥−0.3, only six effective compounds (lignan, β-carotene, β-sitosterol, hederagenin, berberine, and baicalein) were identified from a pool of 22 putative components. Subsequently, molecular docking and dynamics simulation were conducted to verify the accuracy of the predicted bioactive ingredients. The findings indicated that six bioactive ingredients could successfully interact with four key targets (CTNNB1, NFKB1, EGFR, and BCL2). Prior research has demonstrated lignans’ efficacy in safeguarding neuronal cells and enhancing cognitive function (29). EGFR in the CNS maintains neural stem cells, promotes astrocyte and oligodendrocyte maturation, and supports neurite outgrowth (30). Upregulation of EGFR can lead to Aβ42 neurotoxicity and neuroinflammation, while inhibiting EGFR can reduce Aβ plaque deposition and improve cognitive function in AD mouse models (31). In this study, lignan targets EGFR in treating AD, suggesting a potential mechanism for LJF in AD therapy. Increasing evidence suggests that administering β-sitosterol in APP/PS1 mice may decrease Aβ accumulation and enhance cognitive function (32). Previous studies have identified a negative association between cortical BCL2 protein expression and immediate recall memory in individuals with AD, with an observed upregulation of BCL2 protein in the precuneus cortex of these patients (33). This study highlights BCL2 as a key target protein in the therapeutic effects of β-sitosterol for AD, shedding light on the potential mechanism of LJF in treating this condition. β-Carotene, a significant provitamin A compound, possesses an unsaturated hydrocarbon chain with β-rings at both termini (34). CTNNB1, a prominent kinase in tau pathology, exhibits abundant expression within the brain. In addition to its role in regulating tau phosphorylation, the high expression of CTNNB1 is associated with the proteolytic cleavage of the Aβ peptide precursor, specific inhibition of which has been shown to reduce Aβ production through a mechanism involving BACE1 (35). β-carotene has been demonstrated to disrupt apoptotic pathways in the brain, as evidenced by its ability to mitigate the reduction of BCL-2 and the buildup of BAX and caspase-3 in a mouse model of traumatic brain injury (36). Our investigation identified CTNNB1 and BCL2 as crucial protein targets of β-carotene in the context of AD treatment, suggesting a potential mechanistic pathway for LJF in managing AD. In previous studies, hederagenin, a triterpenoid saponin, has been found to exhibit anti-apoptotic, anti-oxidative, anti-inflammatory, and neuroprotective properties (37). Specifically, hederagenin has been found to mitigate oxidative stress and apoptosis in neuronal cells stimulated with Aβ and enhance the degradation of Aβ deposition in APP/PS1 mice (37, 38). NF-KB transcriptional regulation in the BACE1 promoter affects the amyloidogenic process in AD brains by stimulating astrocytes and repressing neuronal cells and non-activated astrocytes, and the NF-KB signaling activation plays a critical role in inflammatory responses, a key characteristic in AD development (39). The current investigation observed that NF-KB serves as the primary target protein for hederagenin in managing AD, suggesting the potential mechanism of LJF in AD treatment. Berberine, classified as an alkaloid, has been demonstrated to effectively treat AD through the modulation of amyloid precursor protein processing, reduction of Aβ secretion, and amelioration of spatial memory deficits in rat models of AD (40). Molecular mechanisms were implicated in inhibiting key pathogenic enzymes, mitigating intracellular oxidative stress, reducing neuroinflammation, inducing autophagy, and preserving neurons from apoptotic cell death (41). In the current investigation, the primary protein targets of berberine in AD therapy were identified as EGFR and BCL2, both of which play essential roles in neuroinflammation and apoptosis, aligning with the proposed mechanism of action. Studies have shown that baicalein, a flavonoid, has important pharmacological effects such as reducing oxidative stress, inflammation, and preventing deposition of amyloid proteins, excitotoxicity, promoting neurogenesis and differentiation, and having anti-apoptotic effects (42). Baicalein improves cognitive function in 3 × Tg-AD mice by modulating activated microglia and reducing neuroinflammation through the CX3CR1/NF-KB pathway (43, 44). Our current results indicate that the principal target proteins of baicalein in AD therapy are NFKB1, EGFR, and BCL2, suggesting baicalein’s underlying mechanism of action in AD treatment. In spite of these, the potential roles of core targets in AD still need to be more critically evaluated based on AD pathology. There is no reason to suspect that they will not be involved in the occurrence of NDs.

Intriguingly, findings of molecular docking and dynamics simulations revealed that β-carotene and berberine exhibit effective binding to MAPK8, while hederagenin demonstrates effective binding to NFE2L2 in this study. MAPK8 is essential for cell processes like proliferation, differentiation, and transcription. Low levels of MAPK8 can lead to reduced BACE1 expression, potentially affecting the amyloidogenic pathway (45). MAPK8 could also activate MAPK signaling pathways which were believed to contribute to AD pathogenesis by inducing neuronal apoptosis and activating β-and γ-secretases (46). Oxidative stress has been strongly implicated in the pathophysiology of AD, antioxidants, particularly those derived from dietary sources, have been proposed as potential agents for the prevention and treatment of AD (47). NFE2L2 is an essential transcription factor that combats oxidative stress in AD by activating antioxidant genes. Hence, it is possible that certain mechanisms are inhibiting the nuclear activity of NFE2L2, potentially leading to neuronal dysfunction (48). As far as we know, there are no reports that compounds of β-carotene, berberine, and hederagenin interact with MAPK8 and NFE2L2, indicating that MAPK8 and NFE2L2 are probable potential new therapeutic targets for LJF against AD. In spite of these, the new findings still need to be for further verification. These new findings provided potential novel targets for AD targeted intervention in the future.

Nevertheless, it is essential to admit the limitations of this study. Current network pharmacology technology struggles to quantify the dose-effect relationship between herbs and diseases, and its network modeling relies on existing databases and experimental data, leading to some compounds and targets being excluded or some potential biases being introduced. In addition, the study using network pharmacology is a static analysis, but disease progression and drug action are dynamic processes. It’s a challenge to find an effective strategy to overcome it. Further *in vivo* or *in vitro* experiments should be validated LJF’s mechanism in treating AD. LJF’s mechanism in treating AD. Despite limitations, the study’s results provide insight into LJF’s effectiveness against AD, with clinical and research value. Of course, potential side effects, toxicity, or long-term safety should also be considered for LJF in the context of AD treatment in future.

## 5 Conclusion

This study indicated that compounds of lignan, β-carotene, β-sitosterol, hederagenin, berberine, and baicalein from LJF mainly regulated core targets of MAPK8, CTNNB1, NFKB1, EGFR, BCL2, and NFE2L2 that involved in AD progression. Our work revealed that LJF effectively treats AD with multi-component and multi-target properties from a systematic perspective. The findings of this study will establish a theoretical basis for the expanded application of LJF against AD and, hopefully, can guide more advanced experimental studies in the future. However, particular *in vitro* and *in vivo* experiments should be performed to validate the computational findings in the future.

## Data Availability

The original contributions presented in the study are included in the article/supplementary material, further inquiries can be directed to the corresponding authors.

## Footnotes

^1^ https://www.rcsb.org/

^2^ https://www.genecards.org/

^3^ https://www.omim.org/

^4^ https://bioinfogp.cnb.csic.es/tools/venny/index.html

^5^ https://david.ncifcrf.gov/

^6^ https://www.string-db.org/

^7^ http://www.alzdata.org

^8^ https://pubchem.ncbi.nlm.nih.gov/

^9^ https://swissmodel.expasy.org/

^10^ https://pymol.org/2/

^11^ https://www.gromacs.org/
